# Home‐based monitoring of falls using wearable sensors in Parkinson's disease

**DOI:** 10.1002/mds.27830

**Published:** 2019-08-26

**Authors:** Ana Lígia Silva de Lima, Tine Smits, Sirwan K. L. Darweesh, Giulio Valenti, Mladen Milosevic, Marten Pijl, Heribert Baldus, Nienke M de Vries, Marjan J. Meinders, Bastiaan R. Bloem

**Affiliations:** ^1^ Department of Neurology Radboud University Medical Center, Donders Institute for Brain, Cognition and Behavior Nijmegen The Netherlands; ^2^ Philips Research, Department Personal Health Eindhoven the Netherlands; ^3^ Department of Epidemiology Erasmus MC University Medical Center Rotterdam Rotterdam the Netherlands; ^4^ Department of Epidemiology Harvard T.H. Chan School of Public Health Boston Massachusetts USA; ^5^ Philips Research North America, Acute Care Solutions Department Cambridge Massachusetts USA; ^6^ Radboud University Medical Center Radboud Institute for Health Sciences, Scientific Center for Quality of Healthcare Nijmegen the Netherlands

**Keywords:** falls incidence, home‐based monitoring, Parkinson's disease, wearable sensor

## Abstract

**Introduction:**

Falling is among the most serious clinical problems in Parkinson's disease (PD). We used body‐worn sensors (falls detector worn as a necklace) to quantify the hazard ratio of falls in PD patients in real life.

**Methods:**

We matched all 2063 elderly individuals with self‐reported PD to 2063 elderly individuals without PD based on age, gender, comorbidity, and living conditions. We analyzed fall events collected at home via a wearable sensor. Fall events were collected either automatically using the wearable falls detector or were registered by a button push on the same device. We extracted fall events from a 2.5‐year window, with an average follow‐up of 1.1 years. All falls included were confirmed immediately by a subsequent telephone call. The outcomes evaluated were (1) incidence rate of any fall, (2) incidence rate of a new fall after enrollment (ie, hazard ratio), and (3) 1‐year cumulative incidence of falling.

**Results:**

The incidence rate of any fall was higher among self‐reported PD patients than controls (2.1 vs. 0.7 falls/person, respectively; *P* < .0001). The incidence rate of a new fall after enrollment (ie, hazard ratio) was 1.8 times higher for self‐reported PD patients than controls (95% confidence interval, 1.6–2.0).

**Conclusion:**

Having PD nearly doubles the incidence of falling in real life. These findings highlight PD as a prime “falling disease.” The results also point to the feasibility of using body‐worn sensors to monitor falls in daily life. © 2019 The Authors. *Movement Disorders* published by Wiley Periodicals, Inc. on behalf of International Parkinson and Movement Disorder Society.

Falling is among the most serious clinical problems faced by older adults, occurring in 19% to 49% of the elderly population.^1^ Falls can have major consequences, such as fractures and other injuries^2^ and have a negative impact on social and psychological well‐being.^3^ Moreover, mortality is increased in individuals with falls.^4^ Parkinson's disease (PD) is a prime example of a progressive neurological condition where falls are common, presumably because many risk factors coincide in this disorder. Specifically, persons with PD have both balance and gait deficits (including freezing episodes) and commonly also cognitive deficits.^5^

Epidemiological studies and evaluations of novel interventions are difficult to design because fall detection in daily life is difficult. The typical methodology for capturing real‐life fall events is the use of diaries.^6^, ^7^, ^8^, ^9^, ^10^ However, diaries have poor reliability, and compliance is suboptimal. Consequently, the outcomes usually correlate poorly with real‐life behavior.^9^, ^10^, ^11^ Modern technology offers new possibilities to overcome those limitations, for example, by using body‐worn wearable sensors.^12^, ^13^, ^14^, ^15^, ^16^ Such sensors can potentially detect falls automatically, quantitatively, and, importantly, continuously in the patient's own environment, thus providing an attractive alternative to self‐reported burdensome and unreliable diaries. Some promising examples of the use of wearable sensors to quantify fall events in controlled settings and free‐living environments were reported in PD.^17^, ^18^ Moreover, sensors can be used together with a personal emergency response system built into the sensor box, thus providing patients with rapid access to emergency assistance, if needed, for example, when they experience difficulty rising after a fall.^19^

In this study, we analyzed data from such a personal emergency response system in a large cohort of elderly participants who used a single wearable falls detector, worn as a necklace, to collect fall events in their own home environment. Using these real‐life data collected for up to 2.5 years of follow‐up, we aimed to determine the hazard ratio of falling among participants with PD when compared with matched elderly persons.

## Methodology

### Study Design and Participants

In this prospective cohort study, we analyzed an existing dataset composed of data from subscribers to a Personal Emergency Response System (PERS–Philips [Cambridge, MA] Lifeline service). No personal, customer, or proprietary data were shared by Philips. This service can provide immediate access to appropriate help. The PERS consists of a device worn as a necklace with multiple embedded sensors (ie, tri‐axial accelerometer and barometer) and is designed to automatically detect fall events in the elderly. It also enables users to press a button to report emergency situations, such as a fall and contact a central response center for help.^19^ When a fall is automatically detected or self‐reported, a call is generated to a central response center and support is provided as needed. In addition to support, the central response center confirms whether a fall event took place. The fall detector can be worn continuously and contains a battery that lasts for more than 18 months.

The study population was extracted from a sample of more than 100,000 subscribers to the commercial PERS service. The service is partially privately paid for by participants and not covered by their health insurance.^20^ To limit sampling bias, all subscribers to the service between January 2012 and June 2014 who subscribed for at least 3 months were included in the eligible cohort. We selected a convenience sample that included all self‐reported PD participants (n = 2063). Using sample characteristics—age, gender, number of self‐reported medical conditions, and domestic conditions (ie, living alone or not)—we extracted a matched control group from those who reported they did not have PD, but who were also prone to falling and had therefore subscribed to the same falls program. No selection based on type of medical condition was applied to the control group (ie, participants included in the control group were subscribers living with a diversity of chronic conditions). For the matching procedure we used the propensity score matching technique, matching cases with a nearest‐neighbor approach guided by logit scores.^21^

The dataset used here is composed of a preexisting dataset of participants whose personal data have been pseudonymized. All participants reported here gave a priori written informed consent based on the terms and conditions of the service. Therefore, additional approval from an external medical ethical committee was not required for this analysis. Note that the analysis was reviewed and approved by the Philips internal board of ethics (Internal Committee for Biomedical Experiments).

### Data Collection and Outcomes

Fall events were reported either by a button‐push or automatically detected by the fall detector worn as a necklace with multiple embedded sensors (ie, tri‐axial accelerometer and barometer). The necklace device uses data from the embedded sensors to identify falls from changes in height, orientation, and impact as experienced during a fall episode. The fall detection algorithm was developed and validated by Philips based on recorded sensor data from approximately 600 simulated falls of 31 healthy volunteers for typical falls (standing, forward, backward, sideward, sitting), from falls using crash dummies for high‐risk situations (eg, stairs) and approximately 30,000 hours of daily‐life activity collected from elderly people.^19^ The results on device performance showed that validity was good, with a detection rate > 95%.^19^ All fall incidents that are reported in this study were confirmed and annotated by a call center.

The dataset was created between January 2012 and June 2014. From this dataset, we extracted data from a window of data of up to 2.5 years after service enrollment. Fall events were collected until the participant was lost to follow‐up or reached the end of the 2.5‐year observational window. Calls to the central response center were initiated either by an automatic fall detection algorithm or a button push by the participant immediately after the fall. False alarms, accidental button presses, or near falls were not labeled as a fall event in the dataset. The database included loggings of all contacts between the participant and the central response center from which the number of falls was determined. Information on demographics and self‐reported medical history, including diagnosis of PD, was collected from all users during a telephone interview conducted by service agents at service enrollment. As for medical history, service agents asked open‐ended questions about which medical conditions participants had and entered the collected information into the database. Information on comorbidities acquired after enrollment and on medication usage were not collected.

The following outcomes were calculated: (1) incidence rate of any fall (ratio between any fall event registered and the observed follow‐up time [falls per person‐year]), (2) incidence rate of a new fall after enrollment (additional hazard ratio of experiencing a new fall event after enrollment for participants having PD in comparison with controls), and (3) 1‐year cumulative incidence of falling (percentage of participants in both groups who had at least 1 fall 1 year after enrollment). For all outcomes, we assessed the difference between PD participants and the matched control group.

### Statistical Analysis

Descriptive analysis was used to report the incidence rate of any fall for both PD participants and the matched control group. Between‐group differences were determined by *t* tests (continuous variables) or χ^2^ tests (categorical variables). We assessed the association of PD with the incidence rate of any fall using analysis of variance models, with PD status, age, gender, and number of medical conditions as independent predictors and number of falls as dependent variables (significance at *P* < 0.05). Using this model as a base, we added 2‐way and 3‐way interaction terms of age, gender, or age × gender with PD in separate sensitivity analyses. We subsequently stratified analyses by age. Similar to our previous work,^22^ we used the median age in the study population to categorize individuals as “middle‐aged” (≤ median age in years) or “old” (> median age in years).

We investigated the association of PD with the incidence rate of a new fall after enrollment using Cox regression models, with PD status, age, gender, and number of self‐reported medical conditions as independent predictors and a new fall after enrollment (yes/no) as the dependent variable. The proportional hazards assumption was verified by plotting the residuals over time (Supporting Information Figure). In separate sensitivity analyses, we added 2‐way and 3‐way interaction terms of age, gender, or age × gender with PD to the main model to assess possible interaction. Finally, a Kaplan‐Meier survival analysis was applied to assess the cumulative incidence of new falls after enrollment during 1‐year follow‐up for both PD and controls.

For all analyses, a *P* value ≤0.05 was regarded as statistically significant. All analyses were performed using R statistical software, version 3.3.2 (R Foundation for Statistical Computing, Vienna, Austria).

## Results

### Participant Characteristics

All 2063 subscribers to the PERS with self‐reported PD were included. A matched group of 2063 subscribers who did not report having PD was considered to be controls. Table 1 lists the participants’ characteristics. Further investigation of participant characteristics on a convenience sample confirmed that the distribution of fall‐related comorbidities was similar between groups (Table 2).

**Table 1:** Characteristics of Parkinson's disease and controls (N = 4126)

Variable	Patients With Parkinson's Disease (n = 2063)	Controls (n = 2063)	*P* Value*
Follow‐up, mean ± SD	1.1 ± 0.6	1.1 ± 0.6	0.9
Age in years, mean ± SD	78.6 ± 8.4	78.4 ± 8.9	0.5
Gender, % of men	48.3	48.1	0.9
Gender, % of women	51.7	51.9	Not tested
Number of medical conditions, mean ± SD	2.6 ± 2.3	2.5 ± 2.2	0.7
Living condition, % living alone	92.1	92.6	0.5

* As a result of the matching procedure, the groups did not differ in any characteristics (*P* > 0.05).

SD, standard deviation.

**Table 2:** Distribution of self‐reported comorbidities (except for Parkinson's disease) associated with an increased fall risk (N = 2184)

Self‐Reported Comorbidity	Controls, n = 1092; n (%)	Parkinson's Disease, n = 1092; n (%)	χ^2^ *P* Value
Cognitive impairment	57 (5.2)	88 (8.0)	0.01
Chronic obstructive pulmonary disease	55 (5.0)	30 (2.7)	0.01
Osteoporosis	17 (1.5)	35 (3.2)	0.1
Diabetes	217 (19.9)	158 (14.5)	0.001
Heart conditions	287 (26.3)	216 (19.8)	0.0004

Data were extracted from a 2.5‐year window, and the average follow‐up was 1.1 years. This average is lower than the window because some participants enrolled later and some participants left the service throughout this period. Reasons for discontinuing the service may have included death, moving to a long‐term facility, or financial reasons.

### Fall Events

A total of 6436 fall events were detected in both groups. We analyzed a multimodal model including self‐reported falls and automatic detected falls. Additional analysis of a subset of subscribers (n = 2184 subscribers) revealed that in 2,425 of the confirmed falls (70%), the button had not been pushed (ie, these fall events were identified only via automatic fall detection). In the remaining 1038 of falls (30%), the events had been reported via button push. This does not exclude algorithm detection, as the algorithm could have detected the fall after the participant pressed the button. In PD patients, the percentage of confirmed falls reported via automatic fall detection was higher than in controls (1891 [73.5%] of falls in PD vs. 534 [60.1%] of falls in controls). Any contact annotated by the call center as being unrelated to an actual fall was excluded from the dataset. The number of false‐positive events was low (4.36 events/person/year in a sample of 2184 participants).

PD participants had a higher incidence rate of any fall in contrast to controls (2.1 vs. 0.7 falls/person‐year, respectively; *P* < 0.0001). The difference in incidence rate of any fall between PD participants and matched participants was more distinct among older participants (Table 3). Among PD participants, 610 (29.6%) registered more than 2 falls during their follow‐up year and were thus classified as recurrent fallers. By contrast, only 300 (14.5%) matched individuals were recurrent fallers (*P* < 0.0001). The absolute number of fall events resulting in emergency transport, but not the proportion, was higher among PD participants (292; 5.9% of all PD falls) in contrast to controls (183; 11.8% of all falls). When only recurrent fallers where analyzed, the number of participants in need of emergency transport was almost double among PD participants (45, 2.2%) than among controls (28, 1.4%).

**Table 3:** Fall incidence for patients with Parkinson's disease and a matched control group

Outcome Measure	Parkinson's Disease Patients, n = 2063	Controls, n = 2063	*P* Value
Incidence rate of any fall			
All participants	2.1	0.7	<0.0001^b^
Falls per person‐years			
Middle aged^a^ (≤78.6 years)	1.7	0.6	<0.0001^c^
Older^a^ (>78.6 years)	2.7	0.8	
Type of faller, n (%)			
Nonfaller	1080 (52)	1425 (69)	<0.0001^d^
Single faller (1 fall/year)	373 (18)	338 (16)	
Recurrent faller (≥2 falls/year)	610 (30)	300 (15)	

a Groups were dichotomized at the mean value (78.6 years).

b Poisson regression.

c Analysis of variance 2‐way interaction analysis.

d Chi‐square test.

### The 1‐Year Cumulative Incidence of Falling

The incidence rate of a new fall after enrollment (ie, hazard ratio [HR] for falling) was 1.8 (95% confidence interval [CI], 1.6–2.0) for PD participants when compared with controls.

**Figure 1:**
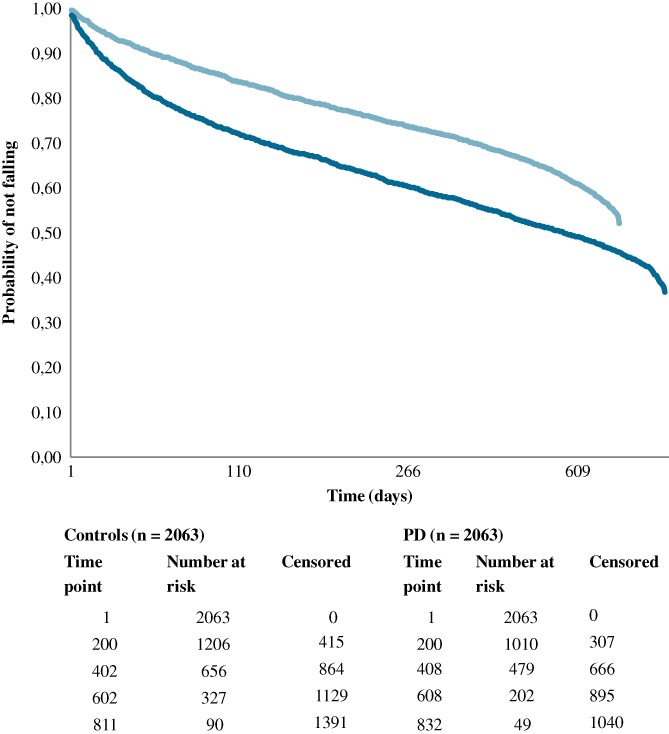
Probability of not falling after enrollment for Parkinson's disease (PD) group (dark blue) and matched elderly participants (light blue). [Color figure can be viewed at http://wileyonlinelibrary.com]

The hazard ratio for falling among PD participants was similar across strata of age (middle‐aged participants, HR = 1.77 and 95% CI, 1.52–2.07; old participants, HR = 1.79 and 2‐way interaction of age by PD *P* = 0.59), gender (in women, HR = 1.69 and 95% CI, 1.44–1.92; in men, HR = 1.89 and 95% CI, 1.64–2.19; 2‐way interaction gender by PD *P* = 0.19), or both age and gender (3‐way interaction term coefficient 1.0, *P* = 0.97). A higher percentage of PD participants had at least 1 fall after a 1‐year follow‐up (983 [or 48% of PD participants] vs. 638, or 31% of controls, *P* < 0.001; Fig. 1).

## Discussion

This large‐scale study determined the real‐life incidence of falls using a wearable system, with all reported falls being confirmed by the faller during a telephone contact immediately after the fall. This enabled us to robustly quantify the HR of falling of PD participants in daily life. The large cohort size allowed us to create both a PD group and a matched control group, leading to an accurate estimation of the additional relative HR in PD. The results from 4126 participants followed on average for 1.1 years showed that PD participants had a much higher incidence rate of any fall when compared with controls. Fall rates were highest for older PD participants, who sustained on average 2.7 falls per person‐year (3 times as often as controls). Finally, PD participants had a 1.8 times higher incidence rate of a new fall after enrollment when compared with controls. This HR was not influenced by the interaction between PD and age or gender.

A high number of fallers among participants with PD has been described previously.^23^, ^24^ A similar prospective cohort study followed 100 PD patients and 55 matched controls.^24^ After 1 year, 54% of PD patients and 18% of controls had experienced falls. Our results (48% fallers among PD patients vs. 31% in matched controls after 1‐year follow‐up) are in accordance. In addition, we show that the interaction of age and PD leads to higher incidence rate of any fall. Specifically, in our cohort, older PD patients fell almost 3 times as often as controls. The effect of age on fall rates was inconsistent in previous reports,^25^ but the aging process may affect the clinical presentation of PD, leading to a worse phenotype.^26^ In addition, older PD patients are likely to have a higher disease duration and greater severity, thus being more prone to falling.^27^

We also show that PD nearly doubles the hazard ratio of a new fall after enrollment. In previous studies,^8^, ^27^, ^28^ the criteria used to select matched individuals may have substantially affected the fall incidence rate as well as the observed hazard ratio for falling associated with PD. For example, Mak and Pang^8^ reported a much higher fall risk rate of 4.2 after following 72 PD patients and 47 controls. However, their control group involved healthy participants recruited from local community health centers who usually have fewer falls. This bias is not present in our study; our PD participants were not selected or excluded based on any medical condition or living style. Thus, we believe that the HR of falling presented here is the most accurate HR for home‐dwelling PD participant subscribers to a personal emergency system. Importantly, this finding confirms that PD is associated with a high incidence rate of falls in daily life, emphasizing the need for fall‐prevention programs tailored to this specific population.

The results of this study are an encouraging example of the feasibility of wearable sensors to monitor falls in real life. In the past years, several initiatives applied wearable sensors for fall detection with good results.^29^ More work is needed to validate accurate algorithms, especially in real life,^30^, ^31^ where reliable ground truth is challenging and achieving high accuracy is ambitious because of confounding daily living activities that can be mistaken as falls. For example, fall detection systems that are based only on an accelerometer may mistake some activities of daily living as falls, leading to suboptimal accuracy in fall detection.^31^ In fact, an accurate and unobtrusive wearable fall monitoring system could improve data collection for trials and support daily care by overcoming the high attrition rates and incorrect data completion seen with paper diaries.^32^ Moreover, wearable sensors have potential to identify patients with a high risk of falling.^33^ Consequently, monitoring with sensors may increase timely referral to falls prevention programs, aiming to decrease the impact of falls on daily life and increase independence.^34^ This potential of sensors becomes more important when considering the higher number of falls in participants with PD resulting in emergency transport that was observed in our study. Future research focusing on refining algorithms for fall detection, fall prediction, and fall risk analysis in daily living should explore the added value of tri‐axial gyroscopes and/or tri‐axial magnetometers. This approached would reduce the false‐positive findings that may plague sensors with only accelerometers^31^ and to thus provide a more robust body of evidence to introduce wearable sensors as instruments for falls monitoring.

### Strengths and Limitations

Beyond the large and well‐matched groups of PD participants and controls, 3 further points strengthen this study. First, this is the first large study to objectively monitor fall episodes during a long follow‐up in a home environment using a wearable sensor. Other smaller initiatives successfully used wearable sensors to collect falls‐related data in the elderly.^35^ However, initiatives to monitor falls for long periods in daily life are scarce. Our study supports the merits of using wearable sensors as an option to objectively and reliably monitor falls in a patient's home environment during a longer period. Second, many prior studies adopted tight inclusion and exclusion criteria,^36^ thus creating a selected population that may not mirror the real population with PD and thus bias the results. Our study applied less tight exclusion criteria, producing a more representative sample of participants with PD in real life. Finally, this study analyzed a total of 6436 fall events that were all confirmed immediately after the incident by a telephone call. This large dataset of confirmed falls ensures that the results reflect the burden faced by participants with PD in real life.

This study also had several limitations. First, all variables, except for the fall episodes, were self‐reported by participants. We could not verify the diagnosis of PD, as this also depended on self‐report. We consider it unlikely that many participants inadvertently reported having PD, whereas in fact they carried a very different diagnosis that was unrelated to any form of parkinsonism. As in any PD study without postmortem confirmation (but perhaps particularly in this study with self‐reported diagnoses), we cannot exclude that some PD participants actually had a form of atypical parkinsonism where falls are generally much more common, thus leading to extra high fall rates. We can also not exclude that some fallers in the control group actually had an early stage of PD that had not yet been identified as such, although falls generally tend to be relatively rare in these early stages of PD.^9^ It would be useful to perform further prospective studies in patients with diagnoses established by experienced clinicians according to accepted international criteria. In addition, future studies could conduct telephone screening of a random set of participants who consent to assess the accuracy of a self‐reported PD diagnosis using validated PD questionnaires.^37^ Second, although all fall episodes were confirmed by an immediate call, the confirmation procedure was only triggered by either algorithm detection or a button press. Therefore, during this process, some fall events may have been missed if the algorithm detection failed, and, at the same time, participants did not use the button press. However, this could only imply that actual fall rates in daily life are even higher than what we observed here, and we have no reason to assume that this false‐negative rate would be different for PD participants and matched controls. Third, the validity of the sensor is based on data from 31 healthy volunteers, not on participants with PD. Although we cannot rule out that the rate of false‐positive findings would be different in PD participants than in healthy volunteers, we believe this is unlikely because fall incidents were confirmed and annotated by a call center using the same methods in both PD participants and controls. We lack separate data on falls detected by algorithm only and on falls registered by a button push. This is because of the fact that if a fall event is labeled as a button push, this does not exclude a simultaneously performed automatic detection as this runs automatically. Therefore, we were unable to make a distinction without overlap between falls reported by button push and by automatic detection. However, we were able to retrieve information on the overall false‐positive rates. The overall number of false‐positive falls is low (4.36 events/person/year in a sample of 2184 participants). It is important to highlight that whether the fall was detected and self‐reported, this procedure did not lead to double registration of the falls because calls were triggered by button push, the same fall could not trigger a second call at the same time. Furthermore, falls that occurred but were missed by the patient or the device were therefore never entered into the database. In addition, although participants were advised to wear the pendant 24/7, we have not recorded data regarding compliance with device usage (ie, actual wear time). From other wearable sensors studies in PD populations, we learned that compliance with sensor usage is on average 68%.^38^, ^39^ Fourth, we acknowledge that the Kaplan‐Meier analysis may overestimate the cumulative incidence in the setting of competing risks.^40^ For example, when studying falls in PD, the use of medications that induce hypotension (eg, a competing risk) is an event that competes with the event of interest. Our analyses were performed in a service‐generated dataset without information on medication or other competing risks for falls. Other types of survival analyses are equally sensitive to this bias in the absence of data on competing risks. In addition, future research would benefit from investigating fall incidence among PERS subscribers who do not have comorbidities. Finally, we acknowledge that our study population is not representative of the general population. Given the population characteristics of our sample, the overall results are primarily applicable to elderly individuals who are prone to falling and are subscribers to a PERS. We cannot exclude that specific demographic or disease‐specific variables (to which we had no access) might have affected the absolute fall rates, but our main analysis focused on the relative difference between a PD population and a matched population without PD, both of whom were similarly motivated and able to subscribe to the falls detection service. So, our main conclusion, namely, that PD is a prime falling disease, stands. It would be interesting to further investigate the fall circumstances (eg, indoors or outdoors, time of day, or even time of year) among PD participants and other elderly by having the fall desk enquire about these each time a fall is reported.

In conclusion, by collecting fall events using wearable sensors, this study demonstrated that having PD nearly doubles the incidence of falling in real life. This confirms that PD is a prime falling disease. In addition, the collection of fall events in more than 4000 participants using a wearable sensor connected to a PERS highlights the potential of using body‐worn sensors for long‐term home monitoring. 

## Author Roles

(1) Research Project: A. Conception, B. Organization, C. Execution; (2) Statistical Analysis: A. Design, B. Execution, C. Review and Critique; (3) Manuscript: A. Writing of the first draft, B. Review and Critique.

A.L.S.d.L.: 1B, 2C, 3B

N.M.d.V.: 1B, 2C, 3B

S.K.L.D.: 2C, 3B

T.S.: 1B, 2B, 2C, 3B

G.V.: 1B, 2B, 2C, 3B

H.B.: 1B, 2B, 2C, 3B

B.R.B.: 1A, 1B, 2C, 3B

M.J.F.: 1A, 1B, 2C, 3B

M.M.: 1C, 2B, 3B

M.P.: 1C, 2B, 3B

## Full financial disclosures of all authors for the previous 12 months

A.L.S.d.L. reports grants from CAPES‐Brazilian Ministry of Education outside the submitted work. N.M.d.V. reports grants from The Netherlands Organization for Health Research and Development outside the submitted work. T.S., G.V., M.P., M.M., and H.B. report working for Philips. B.R.B. reports grants from the Netherlands Organization for Scientific Research, the Michael J Fox Foundation, UCB, Abbvie, Gatsby Foundation, Hersenstichting Nederland Parkinson's Foundation, Verily Life Sciences, Horizon 2020, Topsector Life Sciences and Health, Stichting Parkinson Fonds, Biogen, and from Walk with Path outside the submitted work. S.K.L.D., M.J.F. has nothing to disclose.

## Supporting information


**Figure 3** Residual analysis for probability of falling after enrolment Cox Model
