# Supplementary figures and images for: Home‐based monitoring of falls using wearable sensors in Parkinson's disease
Source: Mov Disord. 2019 Aug 26;35(1):109–15. doi: 10.1002/mds.27830 (PMC7003816; doi:10.1002/mds.27830)

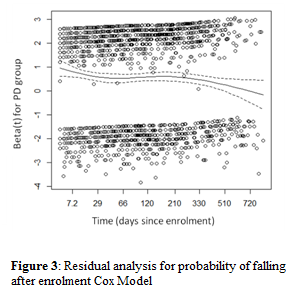

Supplement: Supplementary file 1 — Figure 3 Residual analysis for probability of falling after enrolment Cox Model [file MDS-35-109-s001.tif]
